# Home Is Where the Health Is: Developing Medical Students’ Understanding of Homeless Health

**DOI:** 10.1192/bjo.2024.320

**Published:** 2024-08-01

**Authors:** Lauren Pereira-Greene

**Affiliations:** University College London, London, United Kingdom

## Abstract

**Aims:**

Mental and physical ill-health are both causes and consequences of homelessness. As the cost-of-living crisis forces more people out of their homes, it is imperative that medical students are informed and prepared for this health crisis. Discussions with or about homeless populations are largely absent from the current medical school experience, and are rarely accompanied by homelessness-specific on-the-ground exposure. This project aims to use contemporary literature and the personal experience of a UK medical student to formulate suggestions on how the curriculum can better address homeless health.

**Methods:**

A literature search was performed, including recent work on medical education, inclusion health, and homelessness. Reflection on the author's personal experience at medical school was conducted and compared with existing literature to ascertain validity.

**Results:**

Whilst many students will walk past rough-sleepers on their way into university/hospital, homelessness is a seldom-addressed topic at medical school. In the author's personal experience, there can be a cognitive disconnect between the theoretical principles (e.g. social determinants of health, inclusion health) covered in lectures, and the on-the-ground realities of the isolation, discrimination, and violence that homeless populations face. Since medical students disproportionately come from privileged socioeconomic backgrounds, this disconnect may be due to a lack of exposure underpinned by the assumption that homelessness will never directly affect them.

A review of literature highlighted several worldwide initiatives aiming to develop medical students’ understanding of homelessness. Programmes involved students in health screening, education programmes, and street psychiatry placements. These have been shown to reduce bias and improve student preparedness.

Based on the overlap between literature and the author's own experiences, three focuses for curriculum improvement are proposed: supported exposure, compulsory education, and advocacy. Supported exposure would involve students having formal face-to-face contact with homeless populations, supported by supervision and debriefing. To prepare for these interactions and their potential challenges, students should receive trauma-informed training alongside teaching on inclusion health and social determinants of health. This should be emphasised by medical schools as mandatory, rather than a ‘special-interest’ topic that many students will not engage with. Finally, students should be encouraged to advocate for vulnerable patients both within the clinic, and on a broader systemic level.

**Conclusion:**

This project stresses the urgent need for addressing homelessness within medical education. The proposed focuses aim to cultivate a deeper understanding among medical students about the health challenges faced by homeless populations, fostering empathy and competence in future healthcare professionals.

